# Tibetan Sheep Adapt to Plant Phenology in Alpine Meadows by Changing Rumen Microbial Community Structure and Function

**DOI:** 10.3389/fmicb.2020.587558

**Published:** 2020-10-26

**Authors:** Hongjin Liu, Linyong Hu, Xueping Han, Na Zhao, Tianwei Xu, Li Ma, Xungang Wang, Xiaoling Zhang, Shengping Kang, Xinquan Zhao, Shixiao Xu

**Affiliations:** ^1^Northwest Institute of Plateau Biology, Chinese Academy of Sciences, Xining, China; ^2^Key Laboratory of Adaptation and Evolution of Plateau Biota, Chinese Academy of Sciences, Xining, China; ^3^College of Life Sciences, University of Chinese Academy of Sciences, Beijing, China

**Keywords:** Tibetan sheep, rumen microbiota diversity, rumen microbiota function, phenological periods, adaptability

## Abstract

The rumen microbiota is strongly associated with host health, nutrient absorption, and adaptability. However, the composition, functioning and adaptability of rumen microbiota in Tibetan sheep (TS) across different phenological periods are unclear. In this study we used sequencing of the V4-V5 region of 16S rRNA, qPCR technology and metagenomics to investigate the adaption of rumen microbiota to forage in different stages of phenology. In a grassy period, due to the high nutritional quality of the forage, TS can produce high concentrations of NH_3_-N and short fatty acids by increasing the content of key bacteria in the rumen, such as Bacteroidetes, *Prevotella*, *Succiniclasticum*, *Treponema*, *Butyrivibrio fibrisolvens*, *Fibrobacter succinogenes*, *Prevotella ruminicola*, *Ruminococcus albus*, and *Ruminococcus flavefaciens* to aid in growth. In the withering period, there was a positive correlation between microorganisms which indicated the closely cooperation between microorganisms, and metagenomic analysis showed that the high genes (GHs and CBMs) and subtribe (GH8, GH12, GH45, GH6, GH9, GH5, GH10, GH3, GH52, GH11, GH57, CBM1, CBM4, CBM6, CBM16, CBM37, CBM13, CBM35, CBM42, CBM32, and CBM62) that encode cellulolytic enzymes were significantly increased when the host faced low quantity and quality of forage. Genes involved in metabolic pathways, fatty acid biosynthesis and biosynthesis of antibiotics were significantly enriched, which indicated that rumen microbiota could improve plant biomass deconstruction and energy maintenance in the face of nutritional deficiencies. In the regreen period, both the composition and function of rumen microbiota had obvious disadvantages, therefore, to improve the competitiveness of microorganisms, we suggest TS should be supplemented with high-protein feed. This study is of great significance for exploring the high altitude adaptability of TS.

## Introduction

Plant phenology is cyclical, involving stages such as regreening and blossoming (Wolkovich et al., 2014). In China, plant phenology is an ancient discipline with a history comprising thousands of years (Chu, 1973). The Qinghai-Tibet Plateau (QTP) is known as “the roof of the word.” Changes in plant phenology have significant effects on grassland ecosystems, grazing livestock and the global carbon cycle (Xu et al., 2017b). Due to the high altitude of the QTP, its low levels of oxygen, and changeable climate, the QTP has a growing season of about 100–150 days per year. Herbage turns green in May, grassy green from June to October, and withers in November (Ding et al., 2013). In the grassy period, herbage in natural pasture is plentiful and of good quality, with high levels of protein and carbohydrate (Long et al., 1999), therefore, domestic animals usually fully graze on natural pasture. However, in the plant regreening and withering periods, although some herdsmen stable their livestock in warm sheds for supplementary feeding, the traditional nomadic “living by water and grass” still dominates, and much livestock is maintained on full grazing on natural pasture, which leads to significant weight loss in Tibetan sheep (TS) (12.4–43.7%) and Yaks (25–30%) (Long et al., 2005; Xu et al., 2017a).

The rumen is the first chamber of a ruminant’s stomach, where microorganisms play an important role in decomposing proteins, carbohydrate, starch, sugars, and fats through anaerobic fermentation, and provide nutrients to the host in form of volatile fatty acids (VFAs) and microbial proteins (Huang et al., 2017). The rumen also harbors a dense and diverse microbial community, mainly composed of bacteria, fungi archaea and protozoa (Miron et al., 2001). Bacteria comprise 70% of the microorganisms, playing an important role in the nutrition, immunity and physiological processes of the hosts (Dehority, 2003). The growth of TS has changed due to grazing on natural grassland in different phenological periods, and it is therefore questionable whether the rumen microbiota has changed. To some extent, rumen microbial communities are only temporarily stable (Jami et al., 2013), and they will change with time (Ma et al., 2019), environment (Pitta et al., 2014) and host genetic factors (Han et al., 2020). These factors change the composition and structures of rumen microbial communities, but the functioning of the microbiome may or may not change. If there is no change in microbial function, it is beneficial to maintain the adaptability of the hosts (Ley et al., 2006). However, the microbial function may change, indicating that the microorganisms had functional flexibility (Qin et al., 2020).

To date, the rumen microbial communities of many domestic livestock have been studied by using 16S/metagenomic sequencing. Many uncultured species have been found, indicating their important role in rumen fermentation in organisms like daily cow, cattle, bovine and yak (Jami et al., 2013; Jewell et al., 2015; Xue et al., 2017). TS are the dominant sheep breed, with a population of 50 million on the QTP, and are well adapted to the high altitude, cold, hypoxia, and strong ultraviolet environment of the QTP (Langda et al., 2020). The adaption of TS includes physiological adaptions such a well-developed heart and molecular regulation mechanisms, such as a mutation of EPAS1 which promotes the mean corpuscular hemoglobin concentration and mean corpuscular volume (Wei et al., 2016). However, scant research is available for deep study into the adaptability of TS with respect to rumen microorganisms. In this study, we used 16S rRNA and metagenomic sequencing techniques to characterize the rumen microbial composition and function, and to investigate the adaptability of TS during different phenological periods under full grazing. We hypothesized that due to seasonal variations in forage nutrition, the rumen microbial composition and function would change, and the microbiome in full grazing TS would have different adaption mechanisms in different phenological stages. The results of this study have implications for the understanding of the survival adaptability of TS on the QTP.

## Materials and Methods

### Experimental Site, Design and Sampling Date

The experiment was carried out in the Jiacang ecological animal husbandry professional cooperative of Guinan County, Qinghai province from April 2017 to December 2017. The area has a plateau continental climate with an annual average temperature of 2.3°C, and the herbage growing period is about 120 days. Generally, herbage begins to sprout in May and starts to wither in September. The main grassland type is alpine meadow, and the dominant species are *Kobresia humilis* and *Kobresia capillifolia*. Associated species are mainly members of the Compositae, Ranunculaceae, and Gramineae.

During the experiment, 10 healthy 4-year-old Tibetan male sheep with an average weight of 34.08 ± 2.94 Kg were selected from the same herd and labeled with digital ear markers (Qinghai Jingyi Information Technology Co., Ltd.; type ET-YO) and placed in an alpine meadow for annual grazing. During the forage regreen and grassy periods, TS were grazed on summer-autumn natural pasture (N35°28′∼N35°41′, E 100°42′∼100°54′), and during the forage withering period, TS grazed on winter-spring natural pasture (N35°53′∼35°55′, E 100°74′∼E 100°76′). These 10 TS had no supplementary feeding, none were pregnant, and none were exposed to antibiotics. They had free access to water during the experimental stage.

The forage and rumen fluid were collected in the regreen period (May 2), the grassy period (July 12) and the withering period (December 7). Both herbage and rumen fluid had 10 replicates.

## Forage and Rumen Fluid Collection

The herbage collection methods used in this study were similar to those reported by Ma et al. (2019). Briefly, 10 quadrats (50 cm × 50 cm), with a distance between plots of more than 10 m, were randomly placed in the alpine meadow and the aboveground biomass was collected at each phenological period. The edible forage was collected, dried in a 60°C oven for 24 h, crushed and filtered through a 1 mm sieve for further dry matter nutrition analysis.

The rumen fluid collected methods were similar to those report by Liu et al. (2019). An esophageal tube with a vacuum pump was inserted through the mouth into the rumen for sampling. The rumen content was filtered by four layers sterile gauze, and approximately 30 mL of fluid was collected in each phenological period before the morning grazing. Two milliliters of fluid was stored in liquid nitrogen for later DNA extraction, 5 mL was used for pH measurement, and the rest was used for volatile fatty acid and NH_3_-N determination.

### Determination of Forage Nutrition Composition and Rumen Fermentation Parameters

The dry matter (DM), crude protein (CP), and ether extract (EE) were measured using AOAC methods (Cunniff, 1995). Acid detergent fiber (ADF) and neutral detergent fiber (NDF) were measured using the method described by Soest (Soest et al., 1991). The pH of the ruminal fluid was measured using a portable pH meter (PHSJ-3F). The NH_3_-N content was quantified using a continuous flow analyzer (SEAL Auto Analyzer3, Germany), as described by Russell and Diezgonzalez (1998). The measurement of short volatile fatty acids (SVFAs) was same to that reported by Liu et al. (2019).

### Quantitative Real-Time PCR (qPCR)

Genomic DNA was extracted using E.Z.N.A. stool DAN kits (Omega Bio-tek, Norcross, GA, United States) according to the manufacturer’s protocol. DNA concentration was determined using a NanoDrop 2000 (Thermo Fisher Scientific, Waltham, MA, United States) and the quality was checked using 1% agarose gel electrophoresis. The DNA was amplified using an ABI 7300 Real-Time PCR system (Thermo Fisher Scientific, United States), with fluorescence detection of SYBR® Premix Ex Taq^TM^ II (Tli RNaseH Plus) in a 10 μL reaction system. Amplification consisted of an initial incubation at 95°C for 30 s, followed by 40 cycles of 95°C for 30 s, annealing primer at 55°C for 30 s and extending at 72°C for 30 s. The total microbial DNA was diluted to 1:10 before qPCR assay. The primers used for detection of *Protozoa*, *Butyrivibrio fibrisolvens*, *Fibrobacter succinogenes*, *Prevotella ruminicola*, *Ruminococcus albus*, and *Ruminococcus flavefaciens* are shown in Table 1. The oligonucleotides were synthesized by Takara Biotechnology CO., Ltd., Beijing China. Quantification of bacteria was depicted as a proportion of total rumen bacteria 16S V4-V5 hypervariable genes as follows:

**TABLE 1:** Primers sequences and parameters^a^.

Target	Primer sequence (5′–3′)	Size (bp)	T (°C)	References
*Fibrobacter succinogenes*	5′ GTTCGGAATTACT GGGCGTAAA 3′ CGCCTGCCCCTGA ACTATC	121	60.0	Denman and McSweeney (2006)
*Ruminococcus albus*	5′ CCCTAAAAGCAGTC TTAGTTCG 3′ CCTCCTTGCGGTT AGAACA	176	55.0	Koike and Kobayashi (2001)
*Ruminococcus flavefaciens*	5′ CGAACGGAGATAAT TTGAGTTTACTTAGG 3′ CGGTCTCTGTATGTT ATGAGGTATTACC	132	60.0	Denman and McSweeney (2006)
*Butyrivibrio fibrisolvens*	5′ TCTGGAAACGGA TGGTA CCTTTAAGACAGGAG TTTACAA	295	55.0	Koike and Kobayashi (2001)
*Ruminobacter amylophilus*	5′ CAACCAGTCGCAT TCAGA 3′ CACTACTCATG GCAACAT	642	57.0	Tajima et al. (2001)
*Prevotella ruminicola*	5′ GGTTATCTTGAG TGAGTT 3′ CTGATGGCAACT AAAGAA	485	53.0	Tajima et al. (2001)
*Protozoa*	5′ GCTTTCGWTGGTA GTGTATT 3′ CTTGCCCTCYAAT CGTWCT	223	55.0	Sylvester et al. (2004)

*^*a*^The primer sequences and parameters used in this study referred to the research of Jing et al. (2018). T = optimal PCR annealing temperature.*

$$\begin{array}{c} \mathrm{Relative}\mathrm{quantification}=2^{-\left(\mathrm{target}\mathrm{Ct}-16s\mathrm{V4}\mathrm{V5}\mathrm{Ct}\right)} \end{array}$$

where Ct represents the threshold cycle.

### 16S rRNA Sequencing and Analysis

The DNA extraction, concentration and qualification method was as described above. The DNA was then diluted to 1 ng/μL using sterile water, and stored at −4°C for downstream processing. PCR was performed as described by Ma et al. (2019). Briefly, for each sample, PCR amplification was performed in duplicate using the universal primer 314F/806R, which can amplify the V3-V4 region of the prokaryotic ribosomal RNA gene. The 5′-end of 314F primer included 8 bp unique barcodes to split each sample. After electrophoresis on 2% agarose gels, PCR products with a bright band between 600 and 650 were mixed and purified using AxyPrep DNA Gel Extraction Kits (Axygen Biosciences, Union City, CA, United States) and quantified using QuantiFluor-ST (Promega, United States). The sequencing library was generated using a Qubit® 2.0 fluorometer (Thermo Fisher Scientific) and an Agilent Bio analyzer system. After quality assessment, the library was sequenced on an Illumina HiSeq 2500 platform (Guangzhou Gene Denovo Biotechnology Co., Ltd.), and 250 bp paired-end reads were generated.

To get high clean reads, the raw reads were filtered according to the following rules: reads containing more than 10% unknown nucleotides, and reads containing less than 80% of bases with quality more than 20 were removed. Flash (version 1.2.11) was then used to merge the clean reads as raw tags with a minimum overlap of 10 bp and mismatch error rates of 2% (Magoc and Salzberg, 2011). The raw tags with noisy sequences were filtered using the QIIME pipeline (version 1.9.0) to obtain high-quality clean tags (Caporaso et al., 2010). The clean tags then underwent chimera detection using the UCHIME algorithm to remove all chimera, and effective tags were obtained (Edgar et al., 2011). A total of 2,649,014 effective tags were kept, with over 88,000 for each sample. Filtered sequences were clustered into operational taxonomic units (OTUs) based on a 97% consensus threshold using the UPARSE pipeline (version 7.0.1001) (Edgar, 2013). Then the representative sequences were classified into organisms using the Ribosomal Database Project (RDP) classifier (version v132) (Wang et al., 2007) against the SILVA database (Pruesse et al., 2007).

### Metagenomic Sequencing, Assembly, and Annotation

Fifteen samples (five samples from each phenological period) were selected for metagenomic sequencing. Genomic DNA was extracted using the CTAB method (Porebski et al., 1997). The degree of DNA degradation and potential contamination was measured using 1% agarose gels and quality was measured using a NanoPhotometer® spectrophotometer (IMPLEN, Westlake Village, CA, United States). The concentration was measured using Qubit® dsDNA Assay Kits in a Qubit® 2.0 Fluorometer (Life Technologies, Carlsbad, CA, United States). Using 1 μg DNA per sample as input material, sequencing libraries were generated using NEBNext® Ultra^TM^ DNA library Prep Kits (NEB, United States), and sequences with index codes were added to split each sample. A sonication method was used to generate around 350 bp fragmented DNA, then the DNA fragments were cleaned and extracted using the AMPure XP system. Libraries were prepared on a cBot Cluster Generation System according to the manufacturer’s protocol. After cluster generation, the prepared library was sequenced on an Illumina Hiseq2500 platform (Guangzhou Gene Denovo Biotechnology Co., Ltd.) and 150 bp paired-end reads were generated.

To get high quality clean reads, sequences with more than 50% bases with quality scores lower than 20, or reads with more than 10% unidentified nucleotides were filtered. The reads contaminated by adaptor and sheep hosts reads were removed using Bowite2 (Langmead and Salzberg, 2012). The sequence data were then assembled using MEGAHIT (Li et al., 2015), and k-mers ranged from 21 to 99 were generated for sample-derived assembly, and the unmapped reads of each sample were polled for re-assembly using MEGAHIT, to generate a mixed assembly. Subsequently, overall *de novo* assembly statistics were evaluated by realigning singleton reads using BWA (Li et al., 2015). MetaGeneMark (Zhu et al., 2010) was used to predict the contigs (>500 bp) from each sample, and the Open Reading Frames (ORFs) derived from the assembled contigs were clustered into a non-redundant data set using CD-HIT (Li and Godzik, 2006) with a sequence identify cut-off of 0.95 and 90% read coverage. In order to minimize the number of redundant genes, BWA was used again to count the read numbers which were generated from the re-aligned reads. Finally, a gene catalog was obtained from the non-redundant genes with gene read counts more than two. After read filtering, MetaOthello was used to generate taxonomic profiles. For gene function annotation, all unique ORFs were annotated using DIAMOND (Buchfink et al., 2015) to the KEEG, CAZY and the eggNOG functional database.

### Statistical Analysis

QIIME software (version 1.9.0) was used to analyze the differences in the alpha diversity index. The R software (version 3.5.1) packages “ggplot2” and “reshape2” were used to plot rarefaction curves. The vegan and ggplot2 packages in R were used for principle coordinate analysis (PCoA), Mantel test and Variance partitioning analysis (VPA). The abundance heat map and correlation maps were plotted in R using the packages “pheatmap” and “corrplot.” The phylogenetic molecular networks (pMENS)^1^ were constructed based on random matrix theory (RMT)–based methods (Li H. et al., 2017) and visualized using the Cytoscape software (version 3.3.0). The bar, stack and pie charts were graphed using Origin (version 8.0). SPSS (version 17.0) was used to make single factor ANOVA analysis and Duncan’s multiple test. Effects were considered significant at *P* < 0.05 and results are shown as the mean ± standard error (SE).

## Results

### Forage Nutrition Composition and Rumen Fermentation Parameters Under Different Phenological Periods

As shown in Table 2, the CP (12.13%) and EE (1.84%) contents in the grassy stage were significantly higher than in the regreen and withering stages (*P* < 0.05). In contrast, the ADF content was lowest in the grassy stage and highest in the withering stage (30.21%), and NDF content increased significantly from regreen to withering stage (*P* < 0.05). Rumen fermentation parameters are shown in Table 3. The proportions of NH_3_-N, total SVFAs, isobutyric, butyric, and valeric acids were significantly higher in the grassy stage, whereas acetate and A:P ratio were significantly lower compared to the regreen and withering stages (*P* < 0.01). The withering stage had a significantly increased acetate proportion, but a decreased propionate proportion (*P* < 0.05). Rumen pH showed no significant difference, but appeared to be lower in the grassy stage (*P* = 0.519).

**TABLE 2:** The common forage nutrition composition in different phonological periods (air-dry basis).

Nutrition contents	Phenological periods			*P*-value
	Regreen stage	Grassy stage	Withering stage	
DM^a^	94.240.33^b^	94.600.30^ab^	95.200.25^a^	<0.05
CP	6.320.50^b^	10.130.36^a^	5.180.14^c^	<0.05
EE	0.910.03^bc^	1.840.14^a^	0.510.14^c^	<0.05
ADF	29.820.92^b^	24.520.73^c^	30.210.65^a^	<0.05
NDF	48.311.49^b^	53.011.24^ab^	57.131.68^a^	<0.05

*^*a*^DM, dry matter; CP, crude protein; EE, ether extract; ADF, acid detergent fiber; NDF, neutral detergent fiber. In the same row, values with different letters mean significant different (*P* < 0.05), while with the same or no letter superscripts mean no significant difference (*P* > 0.05).*

**TABLE 3:** The rumen fermentation parameters in the rumen of Tibetan sheep.

Items	Phenological periods			*P*-value
	Regreen stage	Grassy stage	Withering stage	
pH	6.550.09	6.490.04	6.580.05	0.519
ammonium nitrogen (NH_3_-N) (mg/dL)	6.820.51^b^	10.900.75^a^	4.150.51^c^	*P*<0.010
Total short volatile fatty acids (mmol/L)	40.574.14^b^	57.143.47^a^	50.214.14^b^	0.023
Acetate (%)	75.040.29^b^	73.580.01^c^	77.140.32^a^	*P*<0.010
Propionate percentage (%)	15.180.27^a^	15.130.01^a^	14.490.21^b^	*P*<0.010
Isobutyric acid percentage (%)	1.360.03^b^	1.780.01^a^	0.840.04^c^	*P*<0.010
Butyric acid percentage (%)	7.710.32^b^	8.640.57^a^	7.020.26^b^	*P*<0.010
Valeric acid percentage (%)	0.710.03^b^	0.860.05^a^	0.520.01^c^	*P*<0.010
Acetic acid/Propionic acid (A:P)	4.950.10^b^	4.540.09^c^	5.340.09^a^	*P*<0.010

*In the same row, values with different letters mean significant different (*P* < 0.05), while with the same or no letter superscripts mean no significant difference (*P* > 0.05).*

### Rumen Microbiota Profiles

**FIGURE 1:**
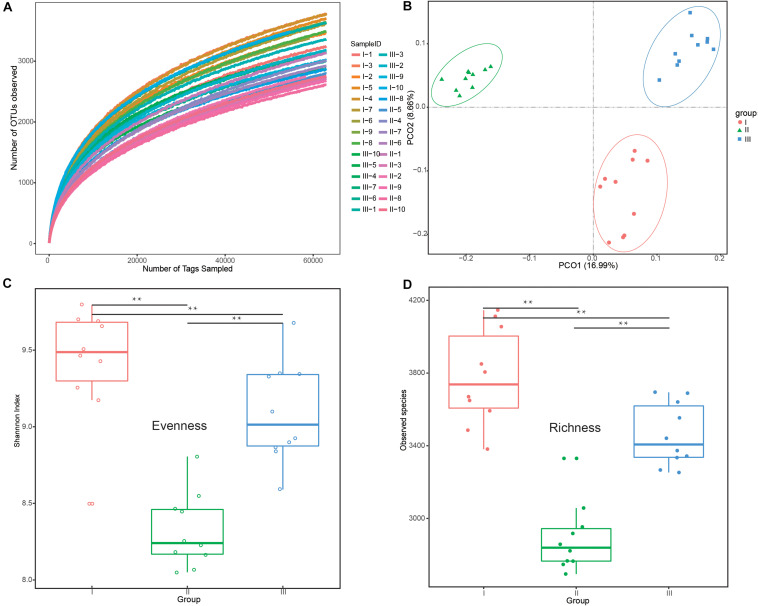
Differences in bacterial diversity and richness under different phenological periods. **(A)** Rarefaction analysis among the 30 different samples at the OTU level. **(B)** Principle coordinate analysis (PCoA) profile of rumen bacteria diversity **(C)** Evenness (Shannon’s diversity index values) at the 3% dissimilarity level. **(D)** Richness (number of observed species) at the 3% dissimilarity level. **P < 0.01 by Tukey test. The groups are regreen stage (I), grassy stage (II), and withering stage (III).

All rarefaction curves of observed OTUs tended to a plateau at 60,000 tags, revealing that the sequencing depth was saturated (Figure 1A). PCoA (Figure 1B) showed clear differences among the groups, which was confirmed by Anosim analysis (*F* = 14.85, *R*^2^ = 0.52, *P* < 0.01). There were a total of 2,699,094 clean reads detected and an average of 89,969 reads via 16S rRNA sequencing (Supplementary Table S1). Of the three phenological periods (Figures 1C,D), the grassy stage had the lowest bacterial diversity in evenness and richness. All indices are shown in Supplementary Table S2.

**FIGURE 2:**
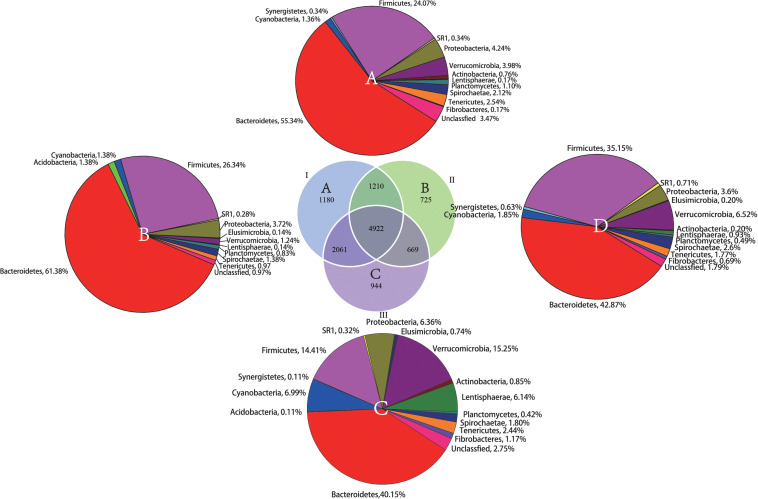
Shared and unique OTUs found in the rumen samples during the different phenological periods. The parenthetical numbers indicate total OTUs proportion in each group, whereas numbers in Venn diagram indicate unique (non-overlapping panels) and shared OTUs (overlapping panels). A total of 11,711 OTUs were detected. **(A)** unique OTUs annotated to the phylum level in the regreen stage, **(B)** unique OTUs annotated to the phylum level in grassy stage, **(C)** unique OTUs annotated to the phylum level in the withering stage. **(D)** The shared OTUs annotated to the phylum level among the three phenological periods.

From the Venn profile (Figure 2), 11,711 OTUs were obtained from 30 rumen samples after data quality control. A total of 4922 (42.03% of the total OTU number) were shared by the phenological stages. Theses OTUs were associated with the phyla Bacteroidetes (42.87%), Firmicutes (35.15%), and Verrucomicrobia (6.52%). The results of annotation of unique OTUs at the phylum level in the grassy stage showed that none of these unique OTUs belong to the phyla Actinobacteri, Firmicutes or Synergistetes. In the withering stage, the unique OTUs were not annotated to Acidobacteria and Elusimicrobia, indicating the unique microbial community composition in different phenological periods.

### Composition of Rumen Microbiota Structure

**FIGURE 3:**
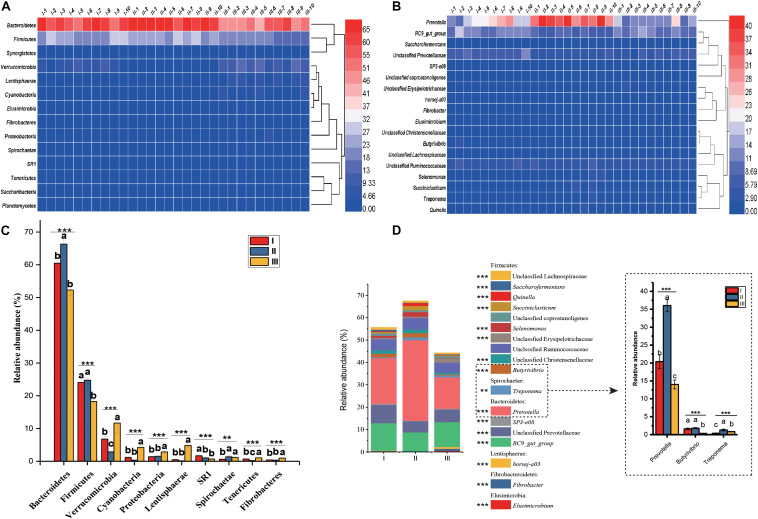
Distribution and significance analysis of rumen bacterial through the 30 samples. Heatmap showing the bacterial relative abundance at phylum **(A)** and genus **(B)** level. The significance analysis of rumen bacteria at phylum **(C)** and genus **(D)** level. The groups are regreen stage (I-1, 2 …10), grassy stage (II-1, 2 …10) and withering stage (III-1, 2 …10). Only the dominant phyla and genus with relative abundance more than 0.5% in one group were listed. Different letters denote statistically significant differences at *P* < 0.01. ^∗∗^*P* < 0.01 and ^∗∗∗^*P* < 0.001 by Tukey test.

In the 30 rumen samples, the dominant phyla were *Bacteroidetes* (59.69%) and Firmicutes (22.34%) (Figure 3A and Supplementary Table S3). Significant shifts were detected in 10 phyla, including Bacteroidetes, Firmicutes, Verrucomicrobia, Cyanobacteria, Proteobacteria, Lentisphaerae, SR1, Tenericutes, Spirochaetae, Fibrobacteres, and Fibrobacteres (*P* < 0.01). Among these phyla, Bacteroidetes and Firmicutes had the highest relative abundance during the grassy stage, but the lowest abundance in the withering stage (*P* < 0.001) (Figure 3C). At the genus level (Figure 3B), *Prevotella* (23.46%) and members of the RC9_gut group (10.75%) were the predominant genera in all samples. Of the 16 genera that changed significantly (Figure 3D and Supplementary Table S4), of these, the relative abundances of *Prevotella*, *Treponema Selenomonas*, *Succiniclasticum*, and *Quinella* were significantly higher in the grassy stage (*P* < 0.01).

### Forage Nutrition Determined Rumen Microbial Diversity

**FIGURE 4:**
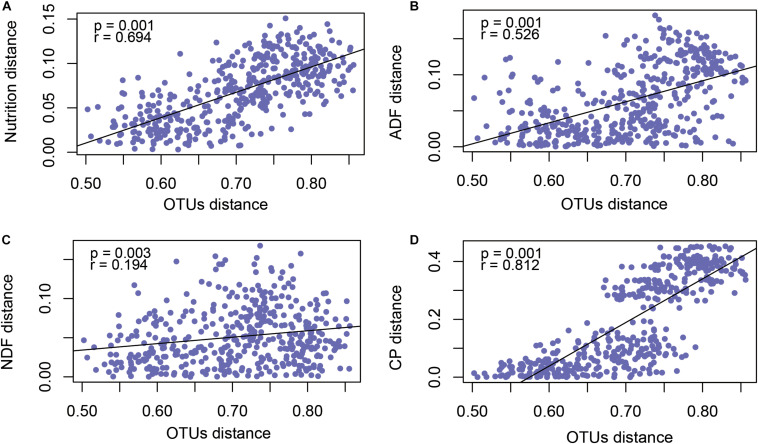
Mantel test revealed the correlation between forage nutrition **(A)**, ADF **(B)**, NDF **(C)**, CP **(D)** and rumen microbiota (OTU level).

In order to explore the factors that affected rumen microflora in different phenological periods, we analyzed the nutrition matrix distance and OTUs matrix distance using a Mantel test, as shown in Figure 4A. The forage nutrition was significantly correlated with rumen microbial community structure (*P* = 0.001, *r* = 0.694). To further determine the forage nutrition factors affecting microbial community structure, we analyzed the correlation between microorganisms and NDF (Figure 4B), ADF (Figure 4C) and CP (Figure 4D). These factors were significantly correlated with the structure of the microbial community, with CP having the greatest impact (*P* = 0.001, *r* = 0.812).

### Rumen Microbial Structure Was Related to Rumen Fermentation Parameters

**FIGURE 5:**
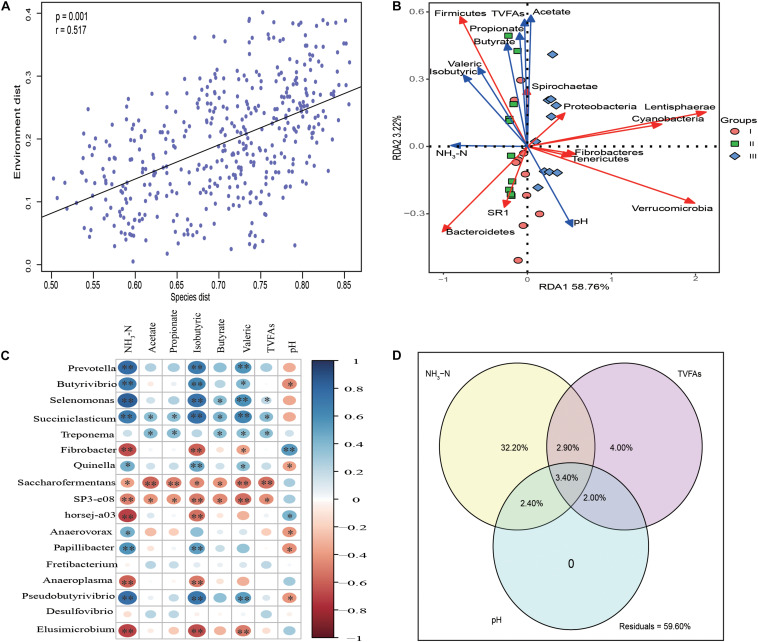
Statistic analysis of the relationship between rumen bacteria community and fermentation parameters. **(A)** Mantel test analysis between bacteria communities (OTU level) and rumen fermentation parameters. **(B)** Redundancy analysis (RDA) of the top 10 phyla in association with rumen fermentation parameters. **(C)** The correlation heatmap using spearman analysis between the top 17 rumen bacteria communities and rumen fermentation parameters. **(D)** Variation partition analysis (VPA) of different factors to the variation of bacteria community structure in rumen samples. The relative abundance of top 17 genera whose relative abundance >0.5% in at least one group are used for spearman analysis and the relative abundance of the genera are used as input for VPA analysis.

Mantel test analysis indicated that the bacterial communities were significantly correlated with rumen fermentation parameters (*P* = 0.001, *r* = 0.517) (Figure 5A). Redundancy analysis (Figure 5B) indicated that the synthesis of NH_3_-N was positively correlated with the abundance of Bacteroidetes. Firmicutes as the second largest phyla was positively correlated with NH_3_-N, acetate, propionate, butyrate, isobutyric, and valeric. Spearman correlation analysis revealed that the genera *Prevotella*, *Butyrivibrio*, *Selenomonas*, *Succiniclasticum*, *Anaerovorax*, *Papillibacter*, and *Pseudobutyrivibrio* were significantly positively correlated NH_3_-N (*P* < 0.05), indicating that these genera were sensitive to the synthesis of NH_3_-N. The levels of fibrolytic bacteria *Prevotella*, *Butyrivibrio*, *Selenomonas*, *Paeudobutyrivibrio*, and the plant polysaccharide degradation bacterium *Treponema*, were significantly positive correlated with various VFAs (*P* < 0.05) (Figure 5C).

Variance partitioning analysis (Figure 5D) showed that 41.4% of the variance in bacterial community structure could be explained by three major variables; NH_3_-N and total volatile fatty acids (TVFAs) could independently explain 32.2, 4% of total variation, respectively. pH had no effect on bacterial community structure. Significant interactions between NH_3_-N and TVFAs (6.30%), NH_3_-N and pH (5.80%) and TVFAs with pH (5.40%) were observed.

### Network Analysis of Bacterial Communities

**FIGURE 6:**
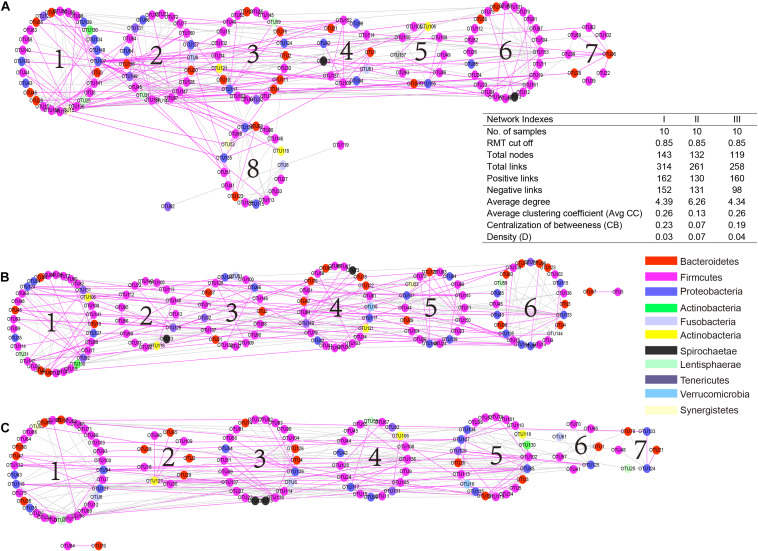
Comparison of rumen bacteria networks during the regreen **(A)**, grassy **(B)**, and withering periods **(C)**. A highly positive correlation is described by *pink color*, whereas highly negative correlation by *green color*. Higher average degree (avg k) and density **(D)** mean a more complex network. A lower average clustering coefficient (avgCC) indicate that bacterial network is mainly composed of relatively isolated nodes. A low level centralization betweenness (CB) signifies a similar bacteria status and more community stability of rumen bacteria. The serial number indicates the number of modules in each group.

To understand the microbial interactions of TS rumen bacterial communities, a bacterial network for each phenological stage was constructed (Figure 6). Using the same threshold (0.85), 314, 261, and 258 links were identified for the hosts in the regreen, grassy, and withering periods, respectively. Notably, in the withering stage, the positive correlations were predominant (160 out of 258), while negative and positive correlations were almost equal in the regreen (152 and 162, respectively) and grassy stages (131 and 130, respectively). We found that the values of average degree and network density in the grassy stage were higher than those in the regreen and withering stages. However, TS in the grassy stage harbored a low level of centralization of betweenness than in the regreen and withering stages.

### Quantitative Analysis of the Major Cellulose Decomposing Bacteria

As shown in Table 4, the number of rumen protozoa in the grassy stage were significantly higher than in the regreen and withering stages (*P* < 0.05). The numbers of *B. fibrisolvens*, *F. succinogenes*, *P. ruminicola*, and *R. flavefaciens* in the grassy stage were significantly higher than that of other two stages (*P* < 0.05).

**TABLE 4:** The relative quantification of ruminal microbes in Tibetan sheep under different phenological periods.

Items	Phenological periods			*P*-value
	Regreen stage	Grassy stage	Withering stage	
*Protozoa*	0.00580.0015^b^	0.04650.0102^a^	0.00280.0005^b^	0.000
*Butyrivibrio fibrisolvens*	0.00430.0008^b^	0.01150.0019^a^	0.00650.0006^b^	0.001
*Fibrobacter succinogenes*	0.00520.001^b^	0.01110.0047^ab^	0.0160.002^a^	0.044
*Prevotella ruminicola*	0.00130.0005^b^	0.01050.0039^a^	0.00230.0008^b^	0.016
*Ruminococcus albus*	0.00020.0001	0.00030.0001	0.00050.0002	0.208
*Ruminococcus flavefaciens*	0.00080.0002^b^	0.00360.0006^a^	0.0010.0001^b^	0.000

*In the same row, values with different letters mean significant different (*P* < 0.05), while with the same or no letter superscripts mean no significant difference (*P* > 0.05).*

### Function of Rumen Bacteria

Based on rumen bacteria diversity and the UniFrac metric (Figure 1B), 15 samples were selected (*n* = 5 each) for metagenomic sequencing. Metagenome sequencing yielded about 14.58G clean bases per sample (Supplementary Table S5), with an average N90 length of 1.65 kb, including 10.5 million non-redundant unigenes and an average ORF length of 580 bp. The number of these non-redundant unigenes reads in the withering stage was significantly higher than in the regreen and grassy stages (*P* < 0.05) (Supplementary Figure S4). Of these unigenes, 10.85% were classified as carbohydrate-active enzymes (CAZy), 30.57% were assigned to KEEG pathways, and 28.59% were classified into eggNOG genes.

### Carbohydrate Genes Related to Cellulolytic Enzymes

To explore the bacterial potential for herbage decomposition during the different phenological periods, we looked for CAZymes in the non-redundant gene sets. A total of 729,643 unique genes were generated after Cazy classification. These genes were assigned to 130 distinct families of GHs, 78 families of GTs, 16 families of CEs, 12 families of AAs, and 73 families of CBMs, as well as 26 families of associated PLs. According to CAZy annotation, there was an increasing trend over the phenological periods in genes encoding GHs, GTs, PLs, CEs, AAs, and CBMs, among which the GHs, PLs, and CBMs in the withering period were present at significantly higher levels than in the regreen and grassy stages (*P* < 0.05) (Supplementary Figure S4 and Supplementary Table S6).

Venn diagrams were constructed for the comparison of CAZyme (Supplementary Figure S1), GHs (Supplementary Figure S2) and CBMs (Supplementary Figure S3), annotated to show the number of proteins either shared or unique to a particular relationship. Overall, 248,129, 231,665, and 251,600 unique CAZyme were annotated in the metagenome of the regreen, grassy, and withering periods, respectively. Of these, 7,023, 8,299, and 11,101 CAZymes were exclusively annotated in respective metagenomes. GHs accounted for 38.81% of the total CAZyme, while 55,624, 50,783, and 56,106 were annotated in the regreen, grassy and withering stags. Of these 2218, 3494, and 3461 GHs were exclusively annotated in respective metagenomes. As with the GHs, the most diverse CBMs (29,097) were annotated in withering metagenomes, with 954 exclusive CBMs. Comparatively high numbers of CAZymes (251,600), GHs (56,106), and CBMs (29,097) along with the presence of elite CAZymes (11,101), GHs (3461), and CBMs (954) indicated the proficiency of the rumen microbiome in the withering stage toward efficient biomass hydrolysis.

**FIGURE 7:**
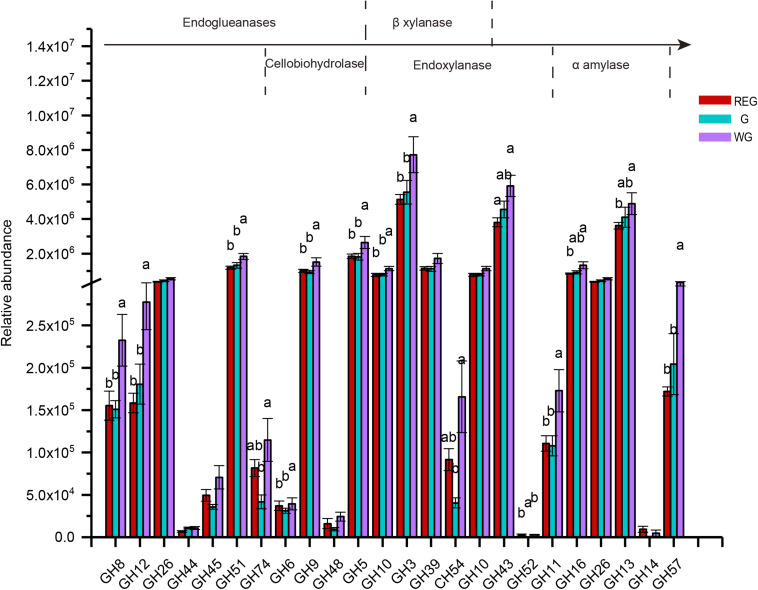
The genes encoding glycoside hydrolases (GH) and the families to which they belong. Different letters denote statistically significant difference at *P* < 0.05.

To further investigate the pivotal cellulose microbial degradation process, we screened for cellulolytic enzymes, including endoglueanases, cellobiohydrolase, β-xylanase, endoxylanase, and α-amylases (Figure 7). In a sequenced-based classification CAZymes of GHs, we found most of genes were enriched in the withering stages, among which GH8, GH12, GH45, GH6, GH9, GH5, GH10, GH3, GH11, GH57 were significantly higher than in the other two stages (*P* < 0.05).

**FIGURE 8:**
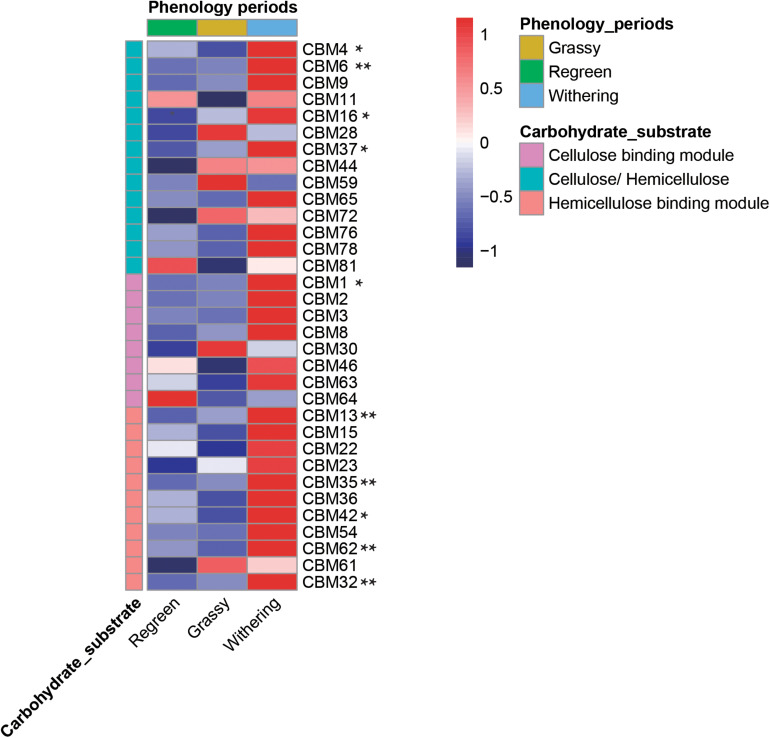
Comparison of CBMs linkd to various cellulose and hemicellulose in rumen metagenome samples during different phenological periods. Heatmap list the abundance of different CBMs. * meat there were significantly difference among groups (*P* < 0.05) and ** meant *P* < 0.01.

CAZymes often exhibited bound carbohydrate known as CBMs, which are vital in the initial stage of crystalline cellulose degradation (Figure 8 and Supplementary Table S7). In total, 14 CBMs which decomposed both cellulose and hemicellulose were annotated in the rumen microbiome. Eight CBMs associated with cellulose and 11 CBMs associated with hemicellulose were annotated. Most of the genes – CBM4, 6, 16, 37, 1, 13, 35, 42, 62, 32 – were highest in the withering stage, decreased in the grassy stage, and lowest in the regreen stage.

### Metabolic Pathway Analysis of Different Phenological Periods

Among the six metabolic pathways, there were no significant differences in metabolism, environmental information processing, organism system or human disease using functional annotation with the KEEG database. The expression of genes related to genetic information processing were significantly lower in the regreen stage (Supplementary Figure S4).

**FIGURE 9:**
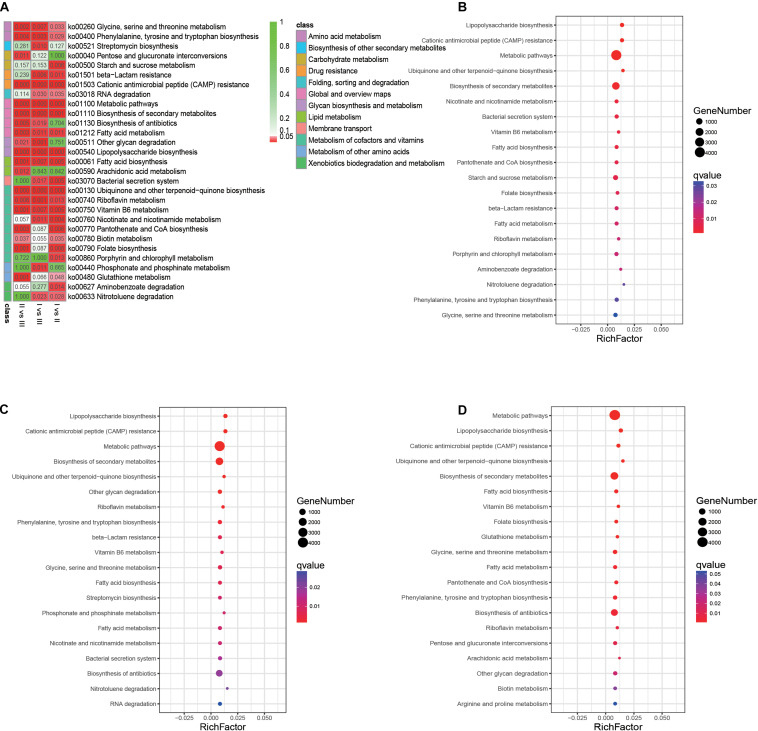
Analysis of metabolic pathways in which genes involved. **(A)** Heatmap showing the significantly different metabolic pathways. **(B)** Gene’s enrichment analysis in the grassy period when compared with the regreen period. **(C)** Gene’s enrichment analysis in the withering period when compared with the regreen period. **(D)** Gene’s enrichment analysis in the withering period when compared with the grassy period. The *q*-value represent the *P*-value corrected by FDR.

A total of 29 pathways with significant differences were identified by pairwise comparison between the three phenological periods (Figure 9A). Enrichment analysis of metabolic pathways in which genes were involved showed that the main metabolic pathways in the rumen of full grazing TS were metabolic pathways and biosynthesis of secondary metabolites, and the enrichment degree was in the following stages: withering period > grassy period > regreen period. The same results were found in genes related to fatty acid synthesis (*q* < 0.01) (Figures 9B–D). The gene enrichment of the antibiotic biosynthesis pathway in the withering period was significantly higher than that of other phenological stages (*q* < 0.01).

## Discussion

### Major Factors Determining Rumen Microbial Diversity

Although rumen microorganisms are relatively stable, they are highly responsive to changes in host genetics (Huang et al., 2017), feeding paradigms (Xue et al., 2017) and diet (Lin et al., 2018; Liu et al., 2019), age (Jami et al., 2013) and environmental factors (Uyeno et al., 2010). Of these, diet was the key factor in determining microbial community structure (Tajima et al., 2001). Ma et al. (2019) studied the rumen microorganism of full grazing yaks and found that the seasonal change in forage composition was the main factor that affected the distribution of microorganisms. In our study, the species, sex, and age of the experimental animals were identical. After removing these factors, we speculated that the significant differences in rumen microbiota structures during different phenological periods would be caused by changes in forage nutrition.

In the grassy period, there was an increased supply of forage, in quality and quantity (Xue et al., 2005), TS could consume forage with high nutrient levels within a small range of activities (Qin et al., 2020). During the regreen and withering periods, in order to obtain more food resources, TS had to seek a wider range of forage with lower nutrition levels. To investigate this hypothesis, we analyzed the correlation between forage nutrition and microbiota. We found that forage nutrition significantly affected rumen microbiota communities, and CP played a major role. Rumen microbial diversity was related to the feeding ecology of the host (Xue et al., 2017). In the grassy period, TS consumed more diverse forage with high nutrition, such as *Poa annua* L, *Stipa capillata* L, *Kobresia humips*, *Kobresia lineata*, and *Leguminosae sp.*, whereas, in the regreen and withering periods, as the biomass of forage decreased and some dominant species were eaten out, high nutrition forage species could not meet the growth demand. TS had to eat broad-leaved forage with poor palatability and nutritional composition (Xiao et al., 2020). We therefore speculated that although food diversity (richness and evenness) indexes of TS in the grassy period were higher than in the regreen and withering periods, due to the differences in feeding niches and forage nutrition structure, the rumen microbiota diversity was lower than in the regreen and withering periods. Li et al. (2016) studied the gut microorganisms of pika living on the plateau and found that the pika could select rare but diverse bacteria in the soil to promote the digestion and absorption of high fiber content forage in the intestinal tract. In our study, due to the low vegetation coverage and the large amount of bare land in alpine meadows during the regreen and withering periods, TS found it easier to access soil from grassland to promote ruminant (McDowell, 1985). Whether or not the increase in the number of soil microorganisms in the rumen caused the increase in the rumen microbial diversity cannot be proven at present, and needs further confirmation.

### Adaptability of Rumen Microbiota in TS on the QTP

Tibetan sheep, a key species in the QTP, have adapted to the harsh plateau environment by changing the morphological characteristics of their organs and physiological-biochemical index (Wei et al., 2016). From the perspective of rumen microbial regulation, Zhang et al. (2016) found that high altitude ruminants, Yak and TS, harbored high levels of *Prevotella spp.* and low levels of *Methanobrevibacter gottschalkii*, which promoted the forage fermentation to produce high levels of acetate, propionate and butyric, and also reduced methane production to avoid energy losses (Shi et al., 2014). Although some researchers have suggested that the unique rumen microbial community composition in TS might relate to their adaptation to high altitude (Huang et al., 2017), little research has been conducted into the adaptability of rumen microorganisms in full-grazing TS using 16S RNA and metagenomic sequencing. In our study, the predominant phyla in the TS rumen were Bacteroidetes and Firmicutes, with average proportions of 59.69 and 22.34%, respectively. The distribution of the main phyla in the rumen were similar to those found in other research into the QTP (Liu et al., 2019; Ma et al., 2019). Kim et al. (2011) have summarized the rumen bacterial distribution of the major domesticated livestock through a meta-analysis of all curated 16S rRNA sequences deposited in the RDP and found the proportion of Bacteroidetes and Firmicutes was about 31 and 56%, respectively. Cunha et al. (2011) investigated the rumen microbiome composition in goats living in semi-arid regions in Brazil and concluded that the proportion of Bacteroidetes and Firmicutes were 37.9 and 56.3%, respectively. Interestingly, in our study, the relative abundance of Bacteroidetes exceeded the mean proportion of Bacteroidetes, while the Firmicutes content was lower than the mean proportion of rumen bacteria that belonged to Firmicutes of the predominant ruminants around the word. The former study showed that members of the phylum Bacteroidetes had ability to efficiently break down proteins and carbohydrate in forage (Huo et al., 2014; Pitta et al., 2014). Therefore, we concluded that the huge number of bacteria in *Bacteroidetes* in plateau ruminants highlights the vital role of these bacteria, which might be beneficial in providing nutrients to the host by degrading the limited resources in the QTP.

### Adaption Strategies of Rumen Microorganisms in Tibetan Sheep at Different Phenological Periods

The rumen microorganisms of TS adapts to the different phenological periods of the QTP. The grassy period is the key stage in which grazing TS gain weight rapidly, due to the high aboveground biomass and profuse nutrition (Sun et al., 2015). Research has shown that *Bacteroidetes* plays an important role in forge nutrition utilization and host disease immunity (Huo et al., 2014; Li Y. et al., 2017; Zhang et al., 2018). *Prevotella* appears to be associated with propionic acid production and plays a pivotal role in degrading and utilizing plant non-cellulosic polysaccharides, protein, starch and xylans (Strobel, 1992; Liu et al., 2019). In our study, the relative abundance of Bacteroidetes and *Prevotella* in the grassy stage provided significant advantages over the regreen and grassy periods. RDA and correlation analysis indicated that the relative abundance of Bacteroidetes and *Prevotella* were positively correlated with the content of NH_3_-N, and the high relative abundance of *Succiniclasticum* and *Treponema* in the grassy period were significant positively correlated with the synthesis of acetate and propionate. Through qPCR analysis, we found that amounts of the main cellulolytic and proteolytic bacteria (Michalet-Doreau et al., 2001), such as *Protozoa*, *B. fibrisolvens*, *P. ruminicola*, and *R. flavefaciens* in the grassy period were significantly higher than that in regreen and withering periods. These results were consistent with the higher dry matter digestibility and growth rate of TS in the grassy period (Sun et al., 2015; Yang et al., 2018). Therefore, we concluded that a higher abundance of functional bacteria in the rumen improves forage digestibility, while producing high concentrations of NH_3_-N and short VFAs to rapidly improve the growth performance of TS in the grassy period.

In September, alpine meadows begin to enter the withering period. With the advance of the withering period, the quality and quantity of forage decreases sharply, especially in winter, which is the most difficult period for TS to survive (Long et al., 1999). In our study, although the relative abundance of Bacteroidetes and Firmicutes in the withering period were lower than in other phenological periods, some phyla of lower abundance, such as Verrucomicrobia, Cyanobacteria, Proteobacteria, Lentisphaerae, Spirochaetae, Tenericutes, and Fibrobacteres were significantly increased. Research has shown that Verrucomicrobia contain a wide range of glycoside hydrolases, which play an important role in the decomposition of polysaccharides and cellobiose (Godoy-Vitorino et al., 2012; Gharechahi et al., 2015). These rumen microbiotas allow TS to obtain energy through the utilization of food, whereas energy compensation strategies permit TS survival in harsh withering period environments.

There are complex interactions among microorganisms, including competition, mutualism, predation, parasitism, and amensalism (Faust and Raes, 2012). Network analysis could shed light upon species interactions according to their co-occurrence (positive) or mutual exclusion (negative) (Li H. et al., 2017). A negative interaction may result in competition or amensalism, while a positive relationship is most likely to lead to mutualism and commensalism (Faust and Raes, 2012). In our networks, in TS in the regreen and grassy periods, the number of positive and negative links was nearly the same, whereas in the withering period positive correlations predominated. We speculated that due to the lack of forage in the withering period, microorganisms could make full use of limited forage resources through increased cooperation. The total number of links in the regreen period were higher than in other periods, indicating that the relationships between microorganisms were closer, and the interactions were more frequent.

A ruminant’s capability to deconstruct plant biomass into fermentable sugars is entirely due to polysaccharide hydrolyzing enzymes produced by rumen microflora (Bohra et al., 2019). The CAZy database provides information concerning all CAZyme families; GHs, GTs, PLs, CEs, CBMs, and AAs, amongst which GHs are the most diverse and abundant group of enzymes, followed by GTs, CBM, CEs, PLs, and AAs (Cantarel et al., 2009; Bohra et al., 2019). GHs are responsible for facilitating the hydrolysis of cellulose (Hess et al., 2011). In our study, the enzymes in GHs family in the rumen metagenome of the withering period showed a more complex process of cellulose degradation. To obtain more information about the GHs family, we classified the families to which GHs belongs, according to Krause et al. (2003). Many genes encoding endocellulase, cellobiohydrolase, endoxylanase, β-Xylosidase and α-Amylase were highly enriched in the rumen microbiota of the withering period. CBMs itself does not exhibit enzymatic activity, but does help in binding GHs to polysaccharides, strengthening their activity (Du et al., 2018; Bernardes et al., 2019). Levels of CBMs in the withering period were significantly higher than in the other two periods. Given these results we speculated that during the long cold season, the genes highly expressed in the rumen microbiota could provide the rumen with the ability to efficiently deconstruct the plant biomass, and promote forage conversion. The functions of the rumen microbiota pathways demonstrate how TS enhance the functions of metabolic pathways, biosynthesis of secondary metabolites, fatty acid biosynthesis and biosynthesis of antibiotics to account for a loss of food quality in the withering period (Kaoutari et al., 2013).

## Conclusion

In this study, the composition and function of rumen microbiota in TS during different phenological periods were compared. We assessed the adaptation strategies of TS using non-invasive methods, laying the foundation for understanding the adaptability of TS in the QTP. Our results indicated that the nutritional quality of herbage, especially the CP content, was the key factor in determining the composition of the rumen microorganisms. Further analysis revealed that rumen microorganisms of grazing TS have varying adaptive mechanisms in different phenological periods. In the grassy period, the high relative abundance of Bacteroidetes, Firmicutes and *Prevotella* promoted forage fermentation to produce a high concentration of NH_3_-N and TVFAs, while some functional flora mainly involved in cellulose decomposition were significantly enriched, potentially increasing plant biomass decomposition and improving the growth of TS. During the withering period, a high abundance of cellulose degrading enzyme genes, more cooperation between microorganisms, and a higher metabolic pathway enrichment could enable TS to make maximum use of the reduced forage resources in winter. During the regreening period, the microorganisms are at a disadvantage in terms of composition and function.

## Data Availability Statement

The datasets presented in this study can be found in online repositories. The names of the repository/repositories and accession number(s) can be found in the article/Supplementary Material.

## Ethics Statement

The animal study was reviewed and approved by Northwest Institute of Plateau Biology, CAS-Institutional Animal Care and Use Committee (OGRD# 2016YFC0501905). Written informed consent was obtained from the owners for the participation of their animals in this study.

## Author Contributions

HL wrote the manuscript. HL, LH, and XH analyzed the data. HL, LM, XW, XiaZ, SK, NZ, and TX contributed to sampling and laboratory. XinZ and SX designed the study and reviewed the drafts of the manuscript. All authors read and approved the final manuscript.

## Conflict of Interest

The authors declare that the research was conducted in the absence of any commercial or financial relationships that could be construed as a potential conflict of interest.

## Footnotes

1 http://ieg4.rccc.ou.edu/mena/

## Supplementary Material

Supplementary Figure S1 jVenn plotting predicted CAZymes that are shared and unique metagenome datasets during the different phenological periods. The histogram below are some statistics based on input lists. The groups are regreen stage (I), grassy stage (II), and withering stage (III).

Supplementary Figure S2 jVenn plotting of predicted GHs that are shared and unique metagenome datasets during different phenological periods. The histogram below are some statistics based on input lists. The groups are regreen stage (I), grassy stage (II), and withering stage (III).

Supplementary Figure S3 jVenn plotting of predicted CBMs that are shared and unique metagenome datasets during different phenological periods. The histogram below are some statistics based on input lists. The groups are regreen stage (I), grassy stage (II), and withering stage (III).

Supplementary Figure S4 **(A)** Comparisons of the total non-redundant genes of rumen microbiomes of TS in regreen, grassy, and withering periods. **(B)** Comparisons of metabolic pathways at KEEG level 1 of TS in regreen, grassy, and withering periods. **(C)** Comparisons of the relative abundance of the CAZymes gene families of the rumen microbiomes of in the rumen of TS in regreen, grassy, and withering periods. Different letters denote statistically significant difference at *P* < 0.05.
