# Supplementary figures and images for: Tibetan Sheep Adapt to Plant Phenology in Alpine Meadows by Changing Rumen Microbial Community Structure and Function
Source: Front Microbiol. 2020 Oct 26;11:587558. doi: 10.3389/fmicb.2020.587558 (PMC7649133; doi:10.3389/fmicb.2020.587558)

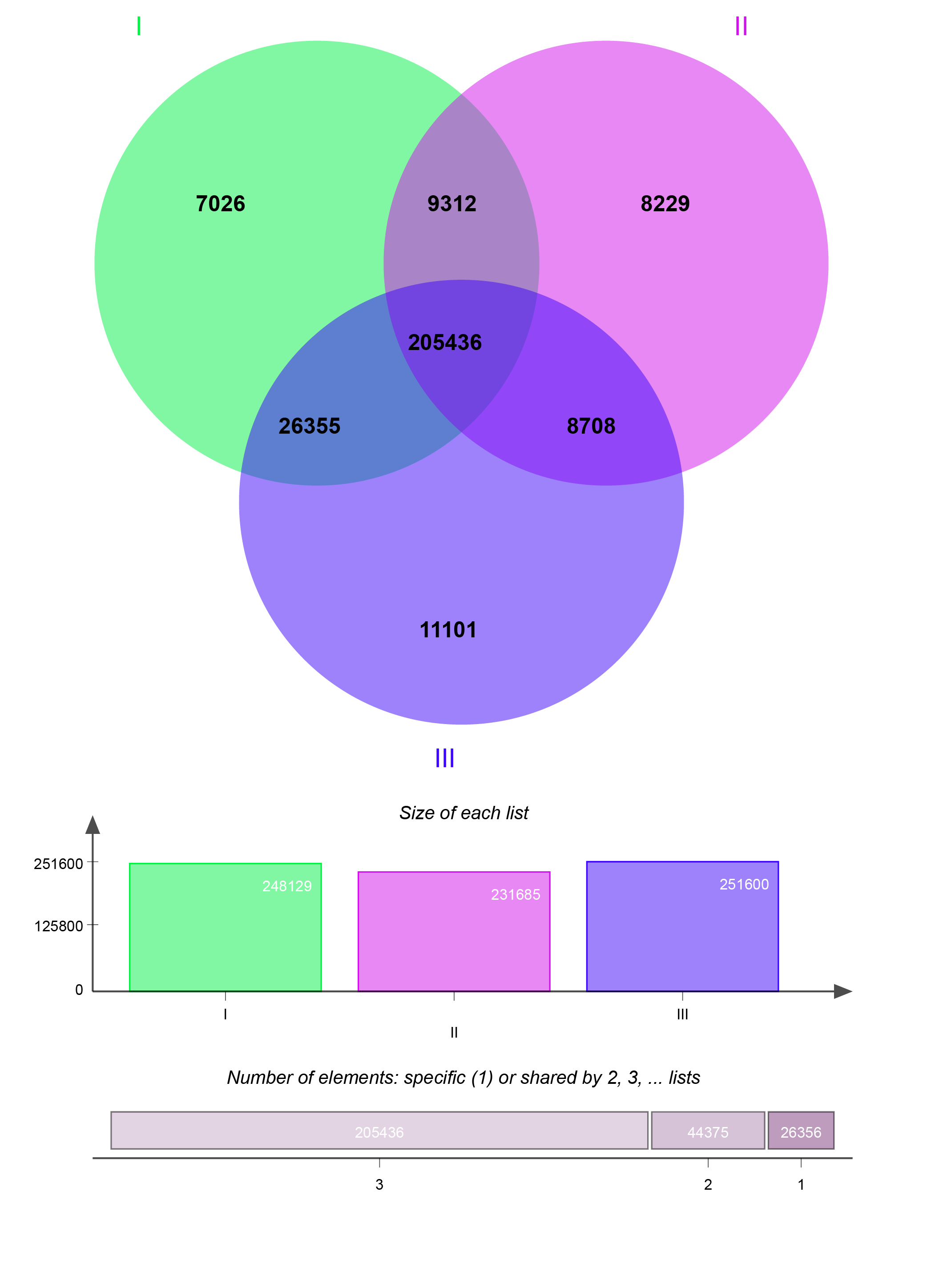

Supplement: Supplementary Figure S1 — jVenn plotting predicted CAZymes that are shared and unique metagenome datasets during the different phenological periods. The histogram below are some statistics based on input lists. The groups are regreen stage (I), grassy stage (II), and withering stage (III). [file Image_1.TIF]

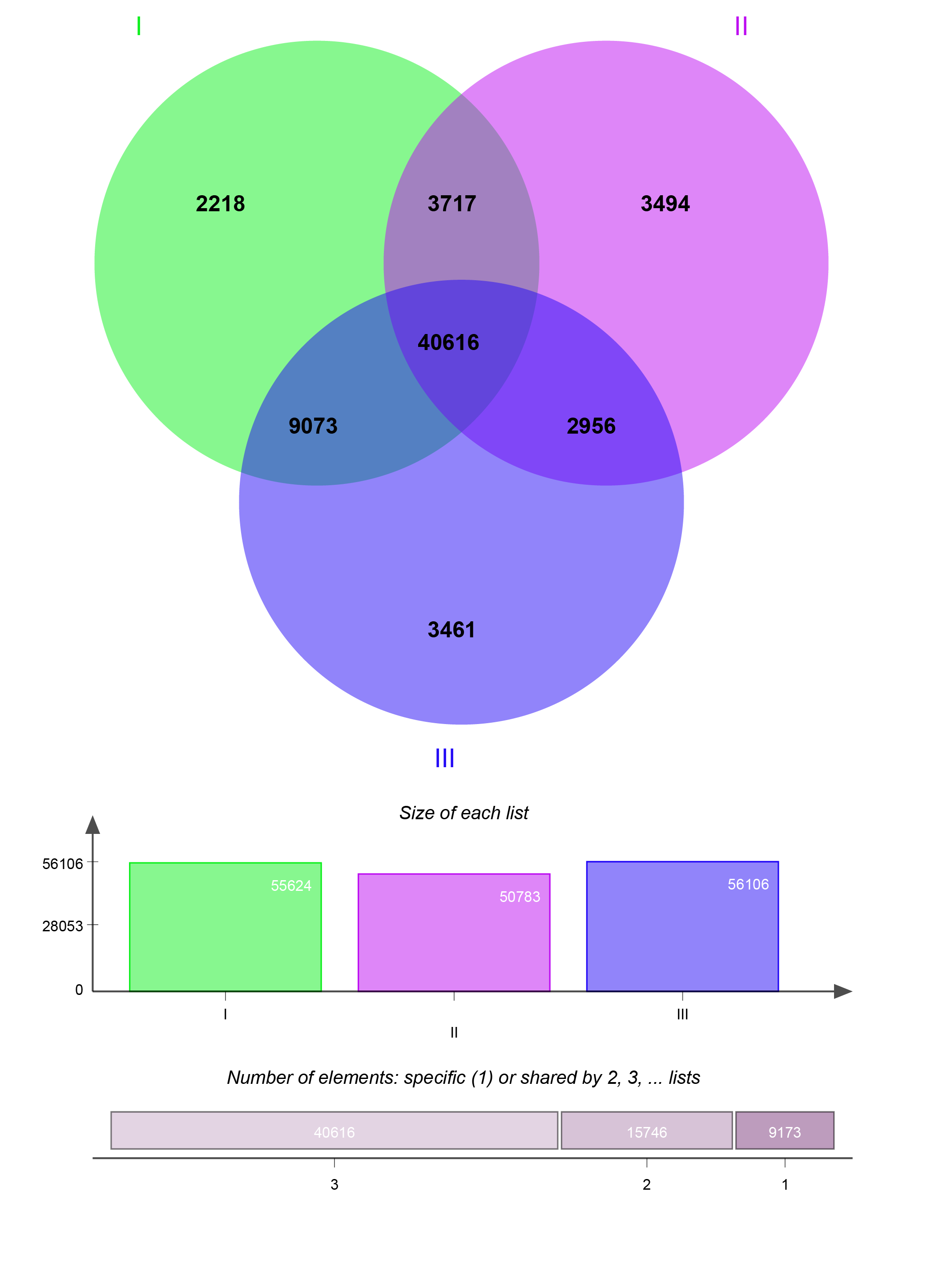

Supplement: Supplementary Figure S2 — jVenn plotting of predicted GHs that are shared and unique metagenome datasets during different phenological periods. The histogram below are some statistics based on input lists. The groups are regreen stage (I), grassy stage (II), and withering stage (III). [file Image_2.TIF]

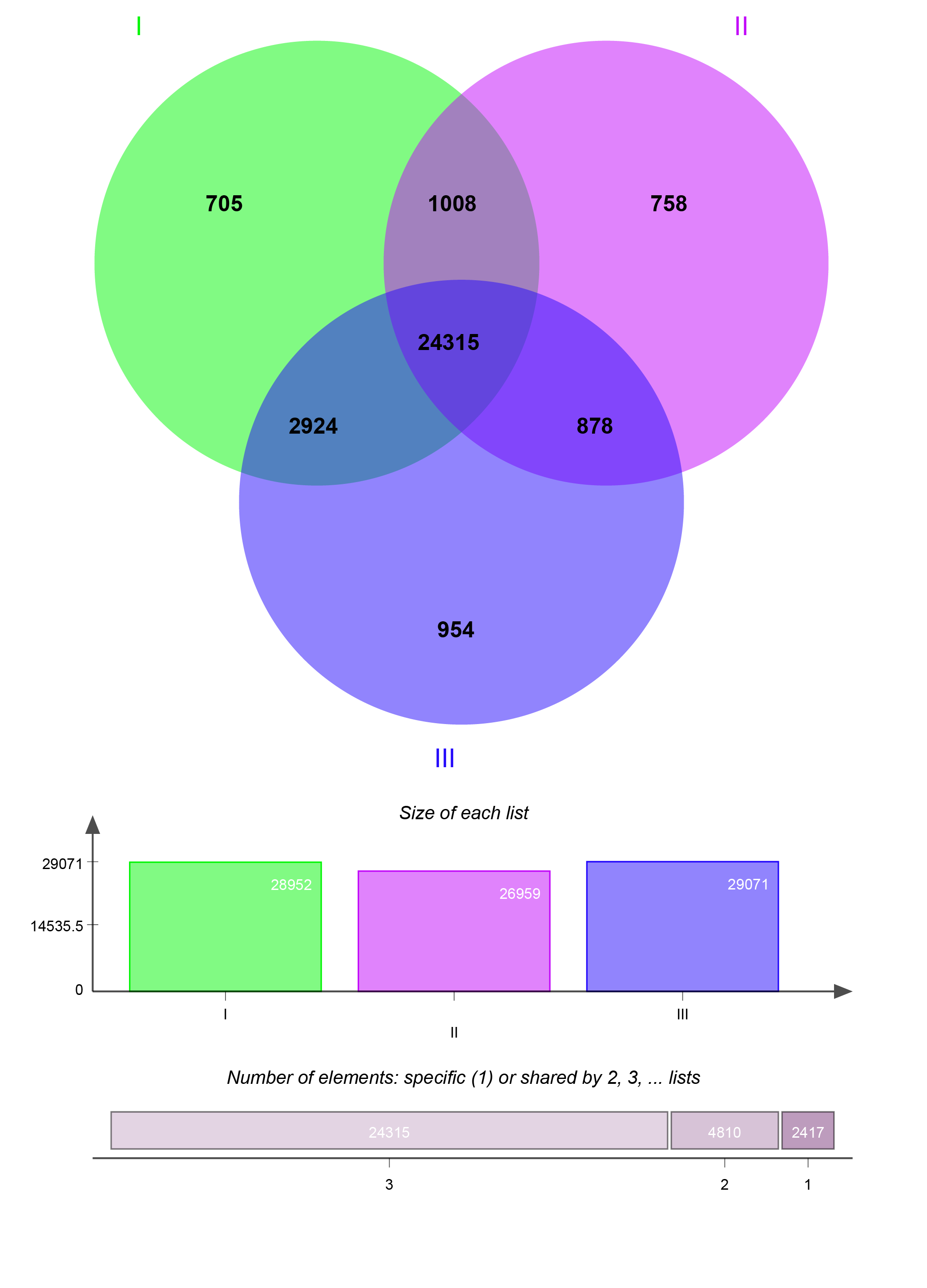

Supplement: Supplementary Figure S3 — jVenn plotting of predicted CBMs that are shared and unique metagenome datasets during different phenological periods. The histogram below are some statistics based on input lists. The groups are regreen stage (I), grassy stage (II), and withering stage (III). [file Image_3.TIF]

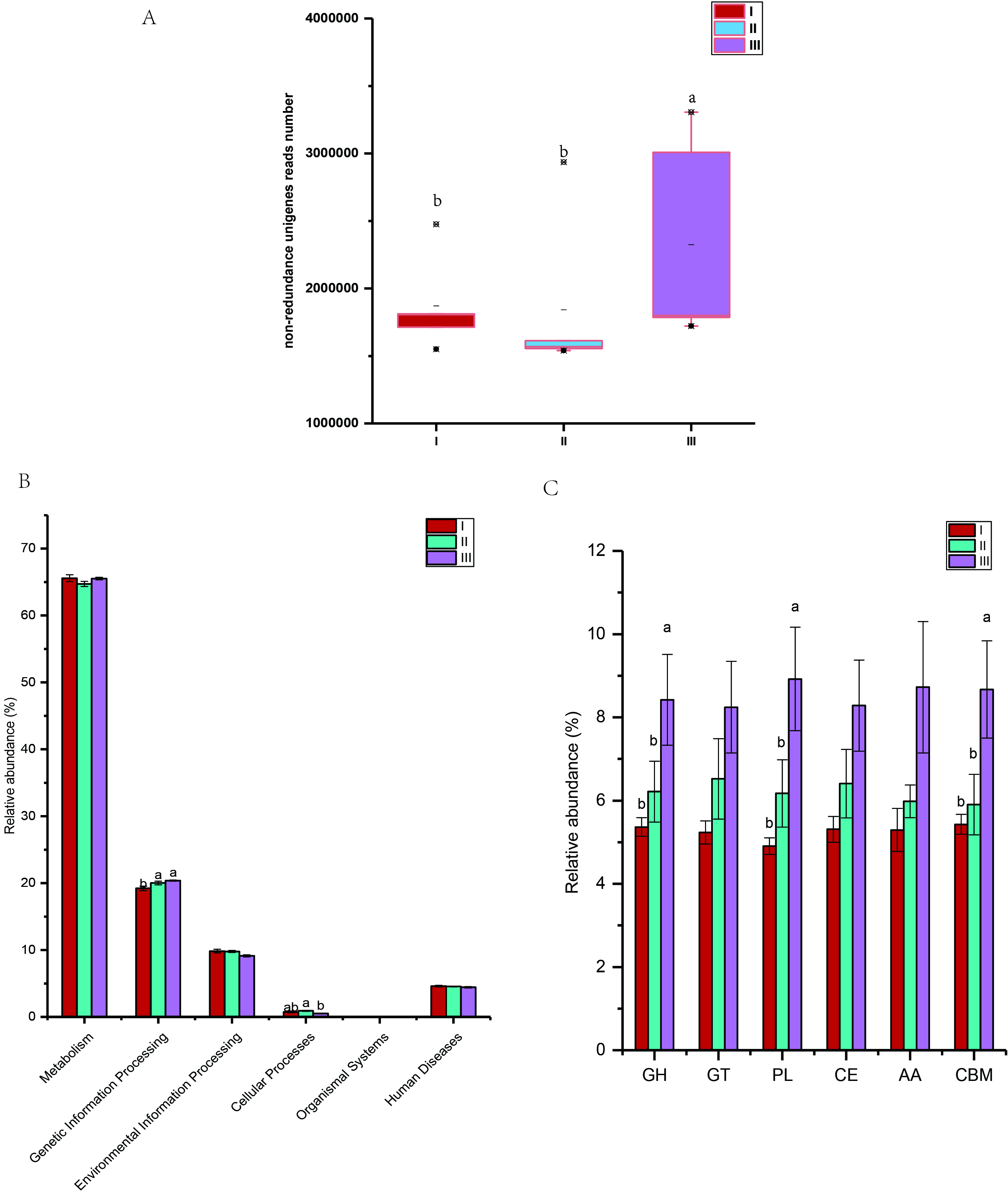

Supplement: Supplementary Figure S4 — (A) Comparisons of the total non-redundant genes of rumen microbiomes of TS in regreen, grassy, and withering periods. (B) Comparisons of metabolic pathways at KEEG level 1 of TS in regreen, grassy, and withering periods. (C) Comparisons of the relative abundance of the CAZymes gene families of the rumen microbiomes of in the rumen of TS in regreen, grassy, and withering periods. Different letters denote statistically significant difference at P < 0.05. [file Image_4.TIF]
