# Supplementary material for: Tibetan Sheep Adapt to Plant Phenology in Alpine Meadows by Changing Rumen Microbial Community Structure and Function
Source: Front Microbiol. 2020 Oct 26;11:587558. doi: 10.3389/fmicb.2020.587558 (PMC7649133; doi:10.3389/fmicb.2020.587558)
Supplement: Supplementary file 5 [file Data_Sheet_1.docx]

| Sample Name | Total reads | Clean reads | Effective Ratio (%) | Max length | Min length | N50 | N90 |
| --- | --- | --- | --- | --- | --- | --- | --- |
| I-1 | 99613 | 96171 | 94.81 | 478 | 201 | 461 | 442 |
| I-2 | 103853 | 99960 | 94.51 | 478 | 201 | 461 | 441 |
| I-3 | 107016 | 103459 | 94.77 | 473 | 201 | 461 | 441 |
| I-4 | 100322 | 96998 | 94.86 | 475 | 201 | 461 | 441 |
| I-5 | 84625 | 81529 | 94.76 | 478 | 201 | 461 | 442 |
| I-6 | 71673 | 69285 | 95.05 | 478 | 201 | 461 | 441 |
| I-7 | 122513 | 117997 | 94.45 | 478 | 201 | 461 | 442 |
| I-8 | 90863 | 87473 | 94.54 | 478 | 201 | 461 | 442 |
| I-9 | 77489 | 74721 | 94.84 | 471 | 201 | 461 | 441 |
| I-10 | 66782 | 64308 | 94.71 | 470 | 201 | 461 | 441 |
| II-1 | 93784 | 90276 | 94.06 | 476 | 201 | 461 | 442 |
| II-2 | 74788 | 71887 | 93.69 | 477 | 201 | 461 | 442 |
| II-3 | 88789 | 85581 | 94.04 | 476 | 201 | 461 | 442 |
| II-4 | 82049 | 79336 | 94.46 | 472 | 201 | 461 | 442 |
| II-5 | 90407 | 87314 | 94.03 | 477 | 201 | 461 | 442 |
| II-6 | 82729 | 79873 | 94.04 | 475 | 201 | 461 | 441 |
| II-7 | 77552 | 74959 | 94.73 | 471 | 201 | 461 | 442 |
| II-8 | 82094 | 79301 | 94.02 | 478 | 201 | 461 | 442 |
| II-9 | 92024 | 88968 | 94.53 | 475 | 201 | 461 | 442 |
| II-10 | 79629 | 76768 | 93.89 | 469 | 201 | 461 | 442 |
| III-1 | 83259 | 80209 | 94.66 | 471 | 201 | 461 | 442 |
| III-2 | 82378 | 79547 | 95 | 477 | 201 | 461 | 441 |
| III-3 | 81577 | 78555 | 94.79 | 478 | 201 | 461 | 442 |
| III-4 | 115727 | 111595 | 95.08 | 478 | 201 | 461 | 442 |
| III-5 | 89161 | 86126 | 95.26 | 478 | 201 | 461 | 441 |
| III-6 | 129216 | 124285 | 94.83 | 473 | 201 | 461 | 441 |
| III-7 | 125940 | 121058 | 94.71 | 475 | 201 | 461 | 442 |
| III-8 | 109988 | 105804 | 94.88 | 468 | 201 | 461 | 442 |
| III-9 | 99063 | 95453 | 95.15 | 477 | 201 | 461 | 442 |
| III-10 | 114249 | 110298 | 95.3 | 478 | 201 | 461 | 442 |
| **Total number** | 2799152 | 2699094 | * | * | * | * | * |
| **Average number** | 93305.1 | 89969.8 | 94.615 | 475.2 | 201 | 461 | 442 |

**Supplementary Table S1** The raw data and quality information of every sample. * means no calculation.

| Alpha diversity | Regreen stage | Grassy stage | Withering stage | P |
| --- | --- | --- | --- | --- |
| Chao1 | 5185.02±89.60 | 4378.91±88.02 | 4746.95± | <0.001 |
| ACE | 5161.48±94.36 | 4397.72±75.28 | 4708.51±63.11 | <0.001 |
| Goods coverage | 0.98±0.00 | 0.98±0.00 | 0.99±0.00 | 0.002 |
| Observed species | 3774.70±79.68 | 2890.70±56.74 | 3458.90±51.69 | <0.001 |
| Shannon | 9.42±0.11 | 8.32±0.07 | 9.09±0.10 | <0.001 |
| simpson | 0.99±0.00 | 0.99±0.00 | 0.99±0.00 | <0.001 |

**Supplementary Table Table S2** The alpha diversity indexes based on 16s rRNA gene at 3% similarity.

**Table S3** Effect of different phenological periods on the relative abundance (%) of rumen bacteria at the phylum level.

| Phylum | Regreen stage | Grassy stage | Withering stage | Average | P value |
| --- | --- | --- | --- | --- | --- |
| *Bacteroidetes* | 60.49±1.31 | 66.28±1.2 | 52.3±1.75 | 59.69±1.34 | <0.001 |
| *Firmicutes* | 24.03±1.35 | 24.72±0.8 | 18.28±0.67 | 22.34±0.78 | <0.001 |
| *Verrucomicrobia* | 6.79±0.83 | 2.84±0.29 | 11.65±0.89 | 7.09±0.78 | <0.001 |
| *Cyanobacteria* | 1.21±0.23 | 0.38±0.04 | 4.27±0.43 | 1.95±0.35 | <0.001 |
| *Proteobacteria* | 1.39±0.17 | 1.53±0.34 | 2.85±0.39 | 1.92±0.22 | <0.001 |
| *Lentisphaerae* | 0.46±0.21 | 0.11±0.02 | 4.82±0.64 | 1.79±0.45 | <0.001 |
| *SR1* | 1.71±0.25 | 1.02±0.11 | 0.67±0.14 | 1.13±0.13 | <0.001 |
| *Spirochaetae* | 0.61±0.1 | 1.39±0.26 | 1.19±0.09 | 1.06±0.12 | <0.01 |
| *Tenericutes* | 0.68±0.1 | 0.24±0.04 | 1.05±0.1 | 0.66±0.08 | <0.001 |
| *Fibrobacteres* | 0.42±0.05 | 0.32±0.09 | 0.95±0.1 | 0.56±0.07 | <0.001 |

Only the dominant phyla with relative abundance more than 0.5% in one group were listed

| **Genus** | **Regreening stage** | **Grassy stage** | **Withering stage** | **Average** | **P value** |
| --- | --- | --- | --- | --- | --- |
| *Prevotella* | 20.38±1.93^b^ | 36.02±1.71^a^ | 14.00±1.25^c^ | 23.46±3.32 | <0.001 |
| *RC9_gut_group* | 12.29±0.76^a^ | 8.44±0.34^b^ | 11.14±0.57^a^ | 10.75±1.23 | <0.001 |
| *Unclassfied Prevotellaceae* | 8.12±0.49^a^ | 4.99±0.18^b^ | 5.56±0.47^b^ | 6.22±1.06 | <0.001 |
| *Unclassfied Christensenellaceae* | 1.49±0.13^a^ | 1.64±0.10^a^ | 0.84±0.08^b^ | 1.32±0.29 | <0.001 |
| *Unclsssfied Ruminococcaceae* | 4.99±0.27 | 5.15±0.20 | 4.48±0.24 | 4.87±0.64 | 0.135 |
| *Butyrivibrio* | 1.60±0.23^a^ | 1.80±0.17a | 0.37±0.03^b^ | 1.26±0.18 | <0.001 |
| *Unclassfied Erysipelotrichaceae* | 0.63±0.09^b^ | 0.63±0.13^b^ | 1.96±0.39a | 1.07±0.22 | 0.001 |
| *Selenomonas* | 0.90±0.19^b^ | 2.04±0.30^a^ | 0.10±0.01c | 1.01±0.13 | <0.001 |
| *Unclassfied coprostanoligenes* | 1.05±0.11 | 0.77±0.10 | 0.90±0.06 | 0.90±0.15 | 0.123 |
| *Succiniclasticum* | 0.60±0.25^b^ | 1.94±0.33^a^ | 0.30±0.06^b^ | 0.93±0.35 | <0.001 |
| *Treponema* | 0.40±0.07^c^ | 1.30±0.27^a^ | 0.82±0.08^b^ | 0.84±0.12 | 0.003 |
| *Fibrobacter* | 0.42±0.05^b^ | 0.32±0.10^b^ | 0.93±0.11^a^ | 0.56±0.18 | <0.001 |
| *Quinella* | 0.35±0.09^b^ | 1.30±0.34^a^ | 0.19±0.04^b^ | 0.61±0.12 | 0.001 |
| *Saccharofermentans* | 0.72±0.1^a^ | 0.18±0.02^b^ | 0.34±0.03^b^ | 0.41±0.09 | <0.001 |
| *Unclassfied Lachnospiraceae* | 1.00±0.12^a^ | 0.77±0.1^ab^ | 0.54±0.05^b^ | 2.31±0.11 | 0.008 |
| *SP3-e08* | 0.53±0.07^a^ | 0.19±0.02^b^ | 0.46±0.07^a^ | 0.39±0.04 | 0.001 |
| *horsej-a03* | 0.14±0.07^b^ | 0.02±0.01^b^ | 0.79±0.20^a^ | 0.32±0.05 | <0.001 |
| *Elusimicrobium* | 0.07±0.04^b^ | 0.02±0.004^b^ | 0.54±0.13^a^ | 0.21±0.06 | <0.001 |

**Table S4** Effect of different phenological periods on the relative abundance (%) of rumen bacteria at the genus level.

Only the dominant phyla with relative abundance more than 0.5% in one group were listed.

**Supplementary Table S5** Basic information of the metagenomic sequencing

| Sample ID | Raw Data (Mbp) | Raw Reads | Clean Data （Mbp) | Clean Reads | Clean_Q20 | Clean_Q30 | Clean_GC (%) | Effective (%) | |
| --- | --- | --- | --- | --- | --- | --- | --- | --- | --- |
| I-1 | 11219 | 74792646 | 10925 | 73666692 | 97.43 | 92.27 | 53.47 | | 98.49 |
| I-2 | 11756 | 78376274 | 11297 | 76645448 | 96.28 | 89.83 | 55.25 | | 97.79 |
| I-3 | 12774 | 85157698 | 12484 | 83999350 | 97.60 | 92.63 | 25.92 | | 98.46 |
| I-4 | 12376 | 82506596 | 12068 | 81309992 | 97.41 | 92.19 | 53.11 | | 98.55 |
| I-5 | 14602 | 97349374 | 14282 | 96074798 | 97.80 | 93.08 | 54.49 | | 98.69 |
| II-1 | 11453 | 76354202 | 11151 | 75187032 | 97.12 | 91.55 | 52.24 | | 98.47 |
| II-2 | 12636 | 84239126 | 12410 | 83301924 | 97.94 | 93.33 | 51.93 | | 98.89 |
| II-3 | 12882 | 85877550 | 12653 | 84921598 | 97.85 | 93.12 | 52.58 | | 98.89 |
| II-4 | 13182 | 87882140 | 12911 | 86779184 | 97.73 | 92.87 | 53.04 | | 98.74 |
| II-5 | 22871 | 152472126 | 22185 | 149276556 | 97.29 | 91.51 | 51.86 | | 97.90 |
| III-1 | 25948 | 172985358 | 25438 | 170910458 | 97.42 | 91.75 | 52.84 | | 98.80 |
| III-2 | 23241 | 154937634 | 22744 | 152940426 | 97.22 | 91.36 | 52.27 | | 98.71 |
| III-3 | 15764 | 105092380 | 15481 | 103917226 | 97.90 | 93.26 | 52.06 | | 98.88 |
| III-4 | 13176 | 87838838 | 12930 | 86808086 | 98.02 | 93.55 | 52.29 | | 98.83 |
| III-5 | 15293 | 101953384 | 15006 | 100755692 | 97.80 | 93.02 | 52.03 | | 98.83 |

**Table S6** Effects of different phenologies on the relative abundance carbohydrate-active enzymes genes.

| Cazy | Regreening stage | Grassy stage | Withering stage | P value |
| --- | --- | --- | --- | --- |
| GH | 5.36±0.23^b^ | 6.21±0.73^b^ | 8.42±1.09^a^ | 0.041 |
| GT | 5.23±0.28 | 6.52±0.97 | 8.24±1.10 | 0.083 |
| PL | 4.91±0.20^b^ | 6.17±0.81^b^ | 8.92±1.24^a^ | 0.019 |
| CE | 5.31±0.31 | 6.41±0.82 | 8.28±1.10 | 0.066 |
| AA | 5.29±0.52 | 5.98±0.39 | 8.73±1.58 | 0.068 |
| CBM | 5.43±0.24^b^ | 5.90±0.73^b^ | 8.67±1.17^a^ | 0.031 |

**Supplementary Table S7** The percentage of different CBMs under different phenological periods.

| Carbohydrate substrate | CBM family | Regreen | Grassy | Withering |
| --- | --- | --- | --- | --- |
| Cellulose/ Hemicellulose | CBM4 | 0.54997 | 0.46554 | 0.76717 |
|  | CBM6 | 2.16786 | 2.28062 | 3.73520 |
|  | CBM9 | 0.49270 | 0.52513 | 0.79411 |
|  | CBM11 | 0.00357 | 0.00123 | 0.00364 |
|  | CBM16 | 0.54055 | 0.69941 | 1.05748 |
|  | CBM28 | 0.00257 | 0.00486 | 0.00328 |
|  | CBM37 | 0.21148 | 0.23918 | 0.36230 |
|  | CBM44 | 0.00807 | 0.01408 | 0.01372 |
|  | CBM59 | 0.00325 | 0.00509 | 0.00320 |
|  | CBM65 | 0.00996 | 0.00911 | 0.01691 |
|  | CBM72 | 0.07092 | 0.10671 | 0.09665 |
|  | CBM76 | 0.00078 | 0.00060 | 0.00165 |
|  | CBM78 | 0.00156 | 0.00130 | 0.00308 |
|  | CBM81 | 0.00001 | 0.00000 | 0.00001 |
| Cellulose binding module | CBM1 | 0.01925 | 0.01980 | 0.02889 |
|  | CBM2 | 2.29339 | 2.32036 | 3.09504 |
|  | CBM3 | 0.26262 | 0.25402 | 0.42271 |
|  | CBM8 | 0.00198 | 0.00220 | 0.00357 |
|  | CBM30 | 0.01426 | 0.02689 | 0.01902 |
|  | CBM46 | 0.00132 | 0.00059 | 0.00185 |
|  | CBM63 | 0.00054 | 0.00014 | 0.00121 |
|  | CBM64 | 0.03868 | 0.00395 | 0.01074 |
| Hemicellulose binding module | CBM13 | 0.38579 | 0.42583 | 0.61497 |
|  | CBM15 | 0.00044 | 0.00015 | 0.00132 |
|  | CBM22 | 0.00984 | 0.00582 | 0.01500 |
|  | CBM23 | 0.08073 | 0.09824 | 0.12102 |
|  | CBM35 | 0.97617 | 1.04587 | 1.73585 |
|  | CBM36 | 0.01227 | 0.01009 | 0.01815 |
|  | CBM42 | 0.08880 | 0.06749 | 0.15192 |
|  | CBM54 | 0.52841 | 0.51082 | 0.77199 |
|  | CBM62 | 0.08387 | 0.06717 | 0.17394 |
|  | CBM61 | 0.13494 | 0.27686 | 0.22987 |
|  | CBM32 | 2.50428 | 2.66555 | 4.42442 |
